# Good performance in difficult times? Threat and challenge as contributors to achievement emotions and academic performance during the COVID-19 outbreak

**DOI:** 10.3389/fpsyg.2023.1264860

**Published:** 2023-11-15

**Authors:** Smirna Malkoc, Daniel Macher, Sabine Hasenhütl, Manuela Paechter

**Affiliations:** ^1^Institute for Practical Education and Action Research, University College of Teacher Education Styria, Graz, Austria; ^2^Educational Psychology Unit, Department of Psychology, University of Graz, Graz, Austria

**Keywords:** adverse learning situation, challenge and threat appraisal, achievement emotions, proneness to anxiety, academic self-concept, learning resources, academic performance

## Abstract

**Introduction:**

The COVID-19 pandemic has emerged as one of the most formidable global crises, leading to the disruptions to education systems worldwide and impacting learning attitudes and psychological well-being of various learner groups, including university students. In this context, students’ appraisals of adverse learning situations play a key role. It is not just the learning situation, but rather students’ appraisal of it which impacts their emotions, attitudes, and behaviors in academic context. The aim of the present study was to investigate how university students’ challenge and threat appraisals were related to emotional learning experiences and learning outcomes during the COVID-19 pandemic. Furthermore, the study focuses on the role of personal and external resources for learning in this context.

**Methods:**

Altogether, 428 students, who attended a Psychology lecture at one Austrian university, filled in a questionnaire about their challenge and threat appraisals of learning circumstances during the COVID-19 pandemic, achievement emotions they experienced during this time as well as gender, proneness to anxiety, academic self-concept, and learning resources. Additionally, students’ performance in the examination was recorded.

**Results:**

The structural equation model emphasizes a crucial role of challenge and threat appraisals for students’ achievement emotions in learning and exam preparation during the COVID-19 pandemic. Challenge appraisals were the strongest predictor for pleasant emotions and threat appraisals were strongest predictor for unpleasant emotions. Proneness to anxiety was related to threat appraisal as well as to experience of more unpleasant and, surprisingly, to positive emotions in adverse learning situation. Academic self-concept and learning resources were identified as important resources for learning in adverse learning situation. Unpleasant achievement emotions were directly and negatively related to academic performance and may thus be seen as a critical variable and crucial obstacle to academic performance.

**Discussion:**

The present study provides implications for learning and instructions which could be implemented by universities in order to support learning and learning attitudes among university students in adverse learning situations.

## 1. Introduction

In their academic life, students may encounter difficult circumstances that can impact and impair their learning and academic performance. As we have experienced in recent years, these circumstances can arise from rather unforeseeable external events, which disrupt the regular educational environment on a wide range, an example being wars, earthquakes, floods, or a pandemic. Also, students may face external study-related adversities in their immediate surrounding as well personal ones, i.e., internal problems like attitudes, that are not conducive to learning, anxiety, etc. External and internal adversities rarely exist in isolation - they often occur simultaneously creating a complex interplay that impacts students’ educational journey.

Among recent global crises the COVID-19 pandemic has certainly created one of the largest disruptions of education systems in history, affecting nearly 1.6 billion learners in more than 190 countries and all continents (United Nations, 2020). Learning has changed tremendously. Due to school and university closures as well as social restrictions, students were mostly confined to their homes, where they had to fulfill their psychological and social needs. During this time, university students were considered as a vulnerable population group (Gómez-García et al., 2022). The pandemic had an extensive negative effect on the quality of their life, their life satisfaction and was connected to feelings of anxiety, stress, depression, and partly even post-traumatic stress disorder (Al-Kumaim et al., 2021; Barbosa-Camacho et al., 2022; Gómez-García et al., 2022; Idoiaga et al., 2022). Furthermore, research suggest that academic engagement among university student decreased significantly during the COVID-19 pandemic (Sheedy O’Sullivan et al., 2022; Azzali et al., 2023). Mental health and overall learning and achievement were threatened (Barbosa-Camacho et al., 2022; Di Malta et al., 2022; Heo et al., 2022; Bongelli et al., 2023).

However, not all individuals respond in the same way to difficult life circumstances. The same difficult event can be perceived as a threat by one person and as a challenge by another one. This is also true for adverse learning situations. The appraisal of a learning situation as challenging or threatening is influenced by personal factors (Jones et al., 2009; Tomaka and Magoc, 2021) and related to a range of emotional, physiological, and behavioral outcomes (Blascovich and Tomaka, 1996; Seery, 2011; Uphill et al., 2019) as well as to performance (Putwain et al., 2017; Feldhammer-Kahr et al., 2021).

The present study looked closer into the role of challenge and threat in adverse learning situations during the COVID-19 pandemic and tries to illuminate how university students’ challenge and threat appraisals were related to emotional learning experiences and learning outcomes and which role personal and external, material resources received in the framework of learning-related variables.

## 2. Appraisals of challenge and threat in the context of personal learning variable, emotional experiences, and academic performance

### 2.1. Challenge and threat appraisals

Situations in which individuals actively perform in order to reach a self-relevant goal, e.g., performance situations, as well as potentially stressful situations can be appraised as challenging or threatening. According to the biopsychosocial model of challenge and threat, two types of appraisals of a situation determine, to which degree it will be perceived as challenging or as threatening (Blascovich and Tomaka, 1996; Seery, 2013). Primary appraisals assess the significance of a situation and the challenge or threat it poses to a person (Feldhammer-Kahr et al., 2021). Secondary appraisals assess whether personal resources are sufficient to meet the demands of the situation (Blascovich and Tomaka, 1996; Seery, 2013; Tomaka and Magoc, 2021). In a learning context, individuals will appraise a situation like exam preparation or taking an exam as challenging, if they perceive the goal as important and if they assume that their personal resources can meet the demands. On the other hand, if individuals perceive their personal resources as being insufficient, the situation will be appraised as threatening. Appraisals of personal resources and situational demands occur automatically and can be constantly updated (Seery, 2013).

Challenge and threat appraisals have a wide range of behavioral and emotional consequences (Hase et al., 2019; Uphill et al., 2019; Tomaka and Magoc, 2021). For example, appraisal of challenge during the COVID-19 pandemic among the university students was related to increased psychological growth (Chu et al., 2022). In the academic context, challenge appraisals are related to greater behavioral engagement and better academic performance, while threat appraisals are related to worse academic performance through lower behavioral engagement (Putwain et al., 2017). Yet, in learning contexts attention should be paid not only to what learners achieve, but also to how they feel in a learning situation. Appraisals of achievement situations are also important antecedents of achievement emotions (Artino et al., 2012; Putwain et al., 2016).

### 2.2. Achievement emotions, learning and performance

Learning situations can be accompanied by a broad range of emotions. These learning-related emotions are called achievement emotions and describe the “affective arousal that is directly related to achievement activities or achievement outcomes” (Pekrun et al., 2011, p. 37). Achievement emotions are influenced by cognitive appraisals of a learning situation (Pekrun, 2006). Hence, previous studies have shown that, among higher education students, cognitive appraisals of their own abilities were related to their achievement emotions during the COVID-19 pandemic (Raccanello et al., 2022). Also, Pekrun et al. (2023) regard achievement emotions as major predictors of performance having stronger effects on performance than gender, age, academic ability, and personality traits.

Valence of achievement emotions is a crucial dimension in this context, important for understanding their structure (Pekrun et al., 2023). In terms of valence, achievement emotions can be differentiated into two groups: pleasant (positive) and unpleasant (negative) ones (Putwain et al., 2021; Pekrun et al., 2023). Examples for pleasant achievement emotions are pride related to academic success, enjoyment of competently performing the work, or gratitude for help in a learning situation (Pekrun et al., 2023). Pleasant achievement emotions are positively related to students’ interest in learning, self-regulation of learning, and they foster students’ attention focus (Pekrun et al., 2011) as well as performance in academic contexts (Peixoto et al., 2017; Paechter et al., 2022). Unpleasant or negative achievement emotions reduce cognitive resources for task performance as well as learning motivation and are related to reduced effort, less time spent for learning and avoidance behavior (Pekrun et al., 2011). Also, they are associated with poorer academic performance for both, young students (Peixoto et al., 2017) and university students (Paechter et al., 2022). Anger that academic success cannot be attained, hopelessness in exam situations, and anxiety during learning or when taking an exam are examples for unpleasant achievement emotions (Pekrun et al., 2023).

### 2.3. Internal and external resources as antecedents for challenge-threat appraisals, achievement emotions and academic performance

Learners can draw on different types of resources when preparing for a situation like an exam. These may lie within the person (internal resources) or may be external. Important external resources in a learning situation include learning materials, technical resources, a dedicated learning space, and allocated time for learning (Paechter et al., 2022). Previous research emphasizes the importance of the external resources, such as technical resources or time for applying the knowledge, in the adverse learning situations; this is true for different learner groups in university context (Ramos-Pla et al., 2021, 2023). For example, students who did not have electronic devices, experienced more difficulties in following synchronous classes during the COVID-19 pandemic (Ramos-Pla et al., 2023). External as well as internal learning resources can be predictors of academic performance (Macher et al., 2013; Ziegler et al., 2019). According to the biopsychosocial model of challenge and threat (Blascovich and Tomaka, 1996; Seery, 2013), perception of personal resources as sufficient to meet the situational demands is connected to the appraisal of the particular situation as challenging, rather than threating.

An internal resource is prior knowledge that individuals bring to learning processes. However, not only the objectifiable prior knowledge, but also its subjective appraisal and beliefs concerning one’s own abilities, represent an important resource. Such beliefs relate to the academic self-concept. It can be defined as evaluative self-beliefs regarding one’s skills and knowledge in academic context. These self-beliefs are formed through past experiences in and interpretations of the academic environment and are shaped by means of social comparisons (Marsh et al., 2023). Academic self-concept can be seen as an antecedent in academic contexts which influences motivation, emotions, cognitions (e.g., cognitive appraisals), and behavior (such as performance; Forsblom et al., 2022; Paechter et al., 2022).

However, personal characteristics may not only facilitate but also hinder learning processes. In this context, proneness to anxiety represents an important variable. It constitutes a personality trait that influences learning and performance in academic situations and has implications for appraisal of a learning situation (Macher et al., 2012; Luttenberger et al., 2018b). Individuals with higher proneness to anxiety tend to appraise stressful situations as threatening, they use less efficient learning strategies, and invest a lower level of personal resources (Macher et al., 2012; Gomes et al., 2017). Proneness to anxiety is linked to state anxiety in academic situations as well as to unpleasant achievement emotions (Macher et al., 2012; Paechter et al., 2022; Trassi et al., 2022). Overall, research suggests that female learners show higher levels of anxiety in academic contexts than male learners (Macher et al., 2012; Paechter et al., 2022) and report higher values on negative emotions compared to men (Fierro-Suero et al., 2022). Individuals with greater anxiety proneness often show a poorer academic self-concept (Macher et al., 2012; Brumariu et al., 2022; Zhang et al., 2023). Also, inadequate learning resources may be related to experiencing more anxiety in learning situations (Hoque et al., 2021).

### 2.4. Present investigation

The present investigation focuses on the appraisals of challenge and threat among university students in adverse learning situation during the COVID-19 pandemic. In this context, relationships between antecedents of learning (personal characteristics that learners bring to the learning processes as well as availability of external resources), appraisals of a learning situation as challenge or threat, achievement emotions, and academic performance were investigated by structural equation modeling (SEM). Based on the previous research as well as on theoretical background described above, such as biopsychosocial model of challenge and threat (Blascovich and Tomaka, 1996; Seery, 2013) as well as control-value theory (Pekrun et al., 2002), investigated variables were grouped into

1. antecedents: proneness to anxiety, academic self-concept, learning resources, gender. Previous studies emphasize an important role of these variables in the learning process. They correlate with each other, are connected to affective and cognitive responses in learning situations and are also related to various outcomes in academic context, including academic performance (Marsh and Martin, 2011; Putwain et al., 2016; Ziegler et al., 2019; Hoque et al., 2021; Paechter et al., 2022). Therefore, these variables were considered as antecedents, i.e., as input variables that may come into play at any stage of learning and may influence all of the subsequent variables.
2. challenge and threat appraisals: Learning situations can be described as motivated performance situations in which students actively perform in order to reach a self-relevant goal. In accordance with biopsychosocial model of challenge and threat (Blascovich and Tomaka, 1996; Seery, 2013) such situations can be appraised as challenging or threatening. Hence, challenge and threat appraisals are directly related to the learning situation, influenced by the antecedents for learning (e.g., personal resources) and, as cognitive appraisals, related to emotional experiences (e.g., achievement emotions) in the learning processes (Pekrun, 2006; Artino et al., 2012; Putwain et al., 2016). Challenge appraisals are associated to the anticipation of success, pleasant emotions, and greater behavioral engagement, whereas threat appraisals are associated with anticipation of failure, negative emotions, and lower behavioral engagement (Putwain et al., 2017; Tomaka and Magoc, 2021). Also, research suggest that, during COVID-19, threat appraisal was related to the negative emotions of distress among university students (Diotaiuti et al., 2023). Thus, it can be assumed that challenge and threat appraisals of learning situation during COVID-19 pandemic are directly related to achievement emotions.
3. achievement emotions in the learning situation, i.e., exam preparation: According to control-value theory as well as to research on emotions in academic contexts, achievement emotions are influenced by cognitive appraisals in learning situation (Pekrun, 2006) and are directly related to academic performance (Pekrun et al., 2002; Peixoto et al., 2017; Paechter et al., 2022). Pleasant achievement emotions foster students’ attention focus as well as their interest in learning and are positively related to academic performance (Pekrun et al., 2002; Peixoto et al., 2017). In contrast, unpleasant emotions reduce cognitive resources for task performance as well as learning motivation and are related to poorer academic performance (Pekrun et al., 2002; Peixoto et al., 2017; Paechter et al., 2022).

**FIGURE 1:**
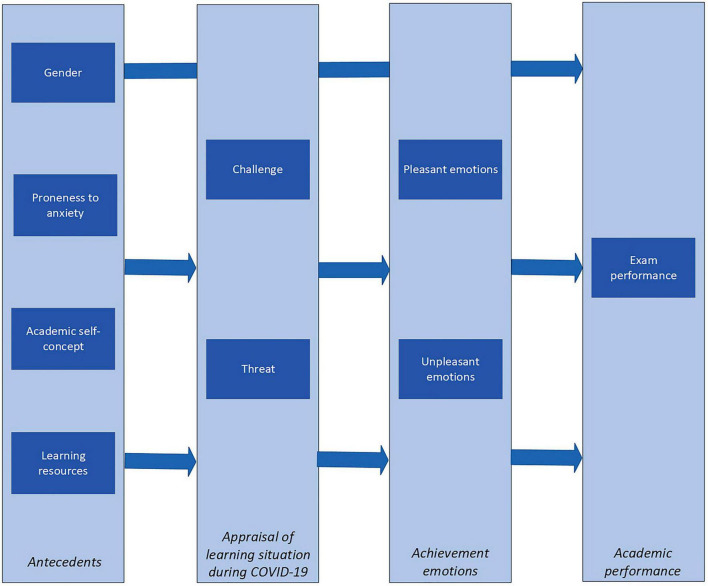
Working model.

Taking into account the relationships described above, a working model has been formulated to explore the connections among the variables (see Figure 1). The current study aims to address the following research questions:

1. How is the appraisal of a specific learning situation during the COVID-19 as challenging or threatening related to achievement emotions and performance?
2. How are achievement emotions related to academic performance?
3. Which role obtain antecedents such as gender, proneness to anxiety, academic self-concept, and learning resources in the appraisal of a particular situation as a challenging or threatening? How are these variables related to achievement emotions and to academic performance?

## 3. Materials and methods

### 3.1. Participants

Data were collected from three lectures on Educational Psychology at an Austrian University. Data collection comprised 428 university students; of them 206 students in the psychology bachelor’s degree (48.1%), 118 students in the psychology master’s degree (27.6%), 83 pre-service teachers (19.4%), and 21 students in other study fields (e.g., pedagogics, human medicine, transcultural communication; 4.9%). All participants attended a course and an exam in one of three lectures of Educational Psychology. The gender composition of the sample with 317 (74.1%) female and 110 (25.7%) male students corresponds to the gender distribution of psychology students as well as pre-service teachers in Austria. Only one participant identified as diverse/non-binary and was excluded from further analyses. Participants were between 18 and 50 years old (*M* = 24.28, *SD* = 4.32). The questionnaire was sent to all students who were to attend an examination in one of the three lectures. Participants filled in a questionnaire 3 days to 1 day before taking their exam. Altogether, 428 participants filled in the questionnaire. Afterward, exam results were recorded. Not all students took part in the exam so that a sample of 314 students had filled in the questionnaire and had taken part in the exam. Participation in the research was voluntary. All participants gave written consent to participate in the study and to confirm that their data were used in an empirical study. The study was performed in the summer semester 2020 and in the accordance with the 1964 Declaration of Helsinki and the American Psychological Association’s Ethics Code. The Ethics Committee of the University Graz had approved the study (GZ. 39/78/63 ex 2016/17).

### 3.2. Measures

*Proneness to anxiety* in academic situations was measured by the Learning and Study Strategies Inventory (LASSI) (Weinstein and Palmer, 2002). This inventory consists of ten subscales with a total of 80 items that have to be rated on a 5-point Likert scale ranging from 1 (not at all typical for me) to 5 (very much typical for me). Only the subscale “proneness to anxiety” was relevant for the present study (item example: “Even when I am well prepared for a test, I feel very anxious.”). The LASSI Anxiety Scale has been selected for several reasons. It is one of the few instruments with items that take into account the study situation in a very descriptive way. In the context of qualification work (Eibl, 2018), validity and reliability analyses had also been carried out in the author team, which spoke in favor of the use of the instrument. Reliability analysis indicated an internal consistency of α = 0.893 (*N* = 427).

*Academic self-concept* was evaluated using three key items from three subscales of the Scales Academic Self-Concept (SASK) instrument. The SASK was developed for and validated in student samples in German-speaking countries with satisfying to excellent internal consistencies (α = 0.74 to α = 0.89; Dickhäuser et al., 2002). Students assessed their abilities and study-related skills compared to the demands of their study and compared to fellow students on a 7-point Likert scale ranging from 1 (low) to 7 (high). Items were “Measured against the demands of my study, I consider my study-related skills to be. (low to high)” (criterion-related self-concept), “I consider my aptitude for my studies…(low to high)” (absolute self-concept), and “With the demands my study places on me I cope.” (less well. to … better than my fellow students). These items had been previously employed in studies examining learning, anxiety, and performance (Macher et al., 2012, 2013). Internal consistency in the present sample was α = 0.817 (*N* = 427).

*Learning resources* were assessed by four items. On a 5-point Likert scale, ranging from 1 (poor) to 5 (very good), students assessed resources in four domains: availability of learning space, temporal resources for learning, access to learning materials and technical resources. These four domains are also assessed in the LIST questionnaire (Learning Strategies in Studies) which was developed for student populations in German-speaking countries (Boerner et al., 2005). An item example is “How well are you equipped regarding learning materials (books, journals, scripts, online documents)?” Internal consistency was α = 0.569 (*N* = 427).

*Appraisal of challenge* and *appraisal of threat* were measured by two scales with four items each. The subscales were developed based on the Challenge-Threat Scale (Drach-Zahavy and Erez, 2002) and an adaption for tertiary education by Feldhammer-Kahr et al. (2021). The two scales had already been employed in a study on teaching during the COVID-19 pandemic in a university context and had yielded satisfactory internal consistency values with Cronbach α > 0.8 (Feldhammer-Kahr et al., 2021). Both, primary and secondary appraisals of challenge and threat were measured. Items example included: “The situation poses a threat to me.” (primary appraisal, threat) and “In general, I think I can cope with this situation.” (secondary appraisal, challenge). Ratings were made on a 5-point Likert scale ranging from 1 (not true) to 5 (true). High values indicated high appraisal of threat respectively of challenge. Reliability indices were α = 0.769 (*N* = 427) for the challenge subscale and α = 0.813 for the threat subscale (*N* = 427).

*Achievement emotions* were measured by the Achievement Emotions Questionnaire (AEQ) (Pekrun et al., 2005). The AEQ comprises three parts which assess achievement emotions, (enjoyment, hope, pride, anger, anxiety, shame, hopelessness, and boredom) experienced in the classroom, in test-related situations, and in learning situations like studying at home, e.g., for exam preparation. The AEQ was initially developed for student samples in the German-speaking countries, but validation studies were extended to other countries like Canada. Studies in external validation show linkages with students’ appraisals, learning, and performance. Studies on internal consistency yielded satisfying to excellent internal consistencies (α = 0.75 to α = 0.92; Pekrun et al., 2011). Only the items on emotions in learning situations were of interest for the present study. Two subscales (which had been investigated and confirmed by factor analysis in other studies; Paechter et al., 2022) were relevant: pleasant and unpleasant emotions in learning situations. Item examples are: “I feel confident when studying.” (pleasant emotions) and “I worry whether I’m able to cope with all my work.” (unpleasant emotions). On the 5-point Likert scale ranging from 1 (did apply very seldom) to 5 (did apply very often), students had to assess how the statements described their achievement emotions experienced in the last week before the exam. Reliability indices measured by Cronbach’s α were α = 0.930 (*N* = 427) for the subscale pleasant emotions (enjoyment, hope, pride) and α = 0.966 (*N* = 427) for the subscale unpleasant emotions (anxiety, hopelessness, anger).

*Academic performance* was measured by the number of points achieved in the final exam. All participants had taken part in one of three courses in Educational Psychology in the summer semester 2020 and in each course, students could take the exam in either June or July 2020. The exams were structured similarly with a length of 45 min exam time and between 22 or 23 multiple-choice or single-choice tasks and two questions with open answers. Exam points achieved by each participant were transformed to z-scores for each exam group; higher values indicate better performance (*N* = 314).

Additionally, demographic variables like gender, age, or study subject were recorded (*N* = 427).

## 4. Results

Descriptive statistics for the investigated variables for the whole sample and separate for women and men are presented in Table 1.

**TABLE 1:** Descriptive statistics for the investigated variables (M, SD, MD, Min, Max, Range) for the whole sample and separate for men and women.

	*M*	SD	Md	Min	Max	Range	*n*
Age	24.28	4.32	23.00	18.00	50.00	18–50	427
Men	24.95	3.74	24.00	19.00	35.00		
Women	24.05	4.50	23.00	18.00	50.00		
Proneness to anxiety	2.47	0.97	2.38	1.00	5.00	1–5	427
Men	2.10	0.91	1.88	1.00	4.75		
Women	2.60	0.96	2.50	1.00	5.00		
Academic self-concept	4.85	0.92	5.00	1.00	7.00	1–7	427
Men	4.95	1.06	5.00	1.00	7.00		
Women	4.81	0.87	4.67	2.00	7.00		
Learning resources	3.89	0.60	4.00	2.00	5.00	1–5	427
Men	3.90	0.57	4.00	2.00	5.00		
Women	3.89	0.61	4.00	2.00	5.00		
Challenge	3.50	0.81	3.50	1.25	5.00	1–5	427
Men	3.55	0.80	3.75	2.00	5.00		
Women	3.48	0.82	3.50	1.25	5.00		
Threat	1.98	0.87	1.88	1.00	4.75	1–5	427
Men	1.87	0.81	1.75	1.00	4.00		
Women	2.02	0.89	2.00	1.00	4.75		
Pleasant emotions	2.84	0.71	2.86	1.06	4.72	1–5	427
Men	2.85	0.76	2.92	1.06	4.72		
Women	2.83	0.70	2.83	1.33	4.67		
Unpleasant emotions	2.16	0.79	1.97	1.00	4.59	1–5	427
Men	1.91	0.72	1.73	1.00	4.00		
Women	2.25	0.79	2.11	1.00	4.59		
Academic performance	0.07	0.95	0.20	−2.52	1.86		314
Men	−0.05	0.92	−0.01	−2.26	1.84		
Women	0.11	0.96	0.27	−2.52	1.86		

Lower numbers indicate lower value in a particular variable/factor. The variable gender is coded as m = 1, w = 0. The variable academic performance has been rescaled using the z-score formula. M, mean; SD, standard deviation; Md, median; min, minimum; max, maximum.

Bivariate correlations for the investigated variables are presented in Table 2.

**TABLE 2:** Bivariate correlations between the investigated variables.

	(1)	(2)	(3)	(4)	(5)	(6)	(7)	(8)
Gender	1							
Proneness to Anxiety	−0.226**	1						
Academic self-concept	0.066	−0.356**	1					
Learning resources	0.014	−0.202**	0.199**	1				
Challenge	0.041	−0.200**	0.208**	0.306**	1			
Threat	−0.075	0.512**	−0.298**	−0.274**	−0.409**	1		
Pleasant emotions	0.015	−0.016	0.214**	0.265**	0.432**	−0.159**	1	
Unpleasant emotions	−0.189**	0.613**	−0.377**	−0.291**	−0.334**	0.607**	−0.264**	1
Academic performance	−0.073	−0.087	0.177**	0.129*	0.063	−0.109	0.014	−0.172**

*p < 0.05; **p < 0.01.

By means of structural equation modeling (SEM) the relationships between variables were investigated. SEM allows to examine direct as well as indirect relationships between resource variables, personal characteristics, gender, cognitive appraisal, and achievement emotions on academic performance. Furthermore, it allows the analysis of the variables of interest at the latent and error-adjusted level (Geiser, 2011; Weiber and Sarstedt, 2021). The data analysis was conducted with MPlus 8.

**FIGURE 2:**
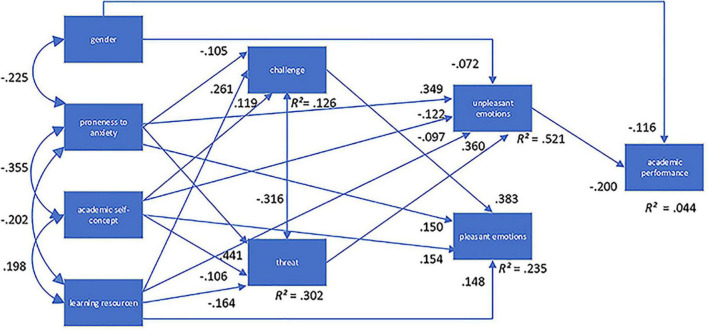
Structural equation model (SEM).

Figure 2 and Table 3 show the model with all significant paths and their corresponding values.

**TABLE 3:** Evaluation of structural model.

Research question	Structural relationship	γ -value
RQ1	Challenge – pleasant emotions	0.383
	Threat – unpleasant emotions	0.360
	Challenge – threat	−0.316
RQ2	Unpleasant emotions – performance	−0.200
RQ3	Gender – anxiety	−0.225
	Gender – unpleasant emotions	−0.072
	Gender – performance	−0.116
	Anxiety – academic self-concept	−0.355
	Anxiety – learning resources	−0.202
	Anxiety – challenge	−0.105
	Anxiety – unpleasant emotions	0.349
	Anxiety – threat	. 441
	Anxiety – positive emotions	0.150
	Academic self-concept – learning resources	0.198
	Academic self-concept – challenge	119
	Academic self-concept – threat	−0.106
	Academic self-concept – pleasant emotions	0.154
	Academic self-concept – unpleasant emotions	−0.122
	Learning resources – challenge	0.261
	Learning resources – pleasant emotions	0.148
	Learning resources – threat	−0.164
	Learning resources – unpleasant emotions	−0.097

Only significant paths are reported.

The χ^2^ test and descriptive fit indices suggest a good model fit (χ^2^[11] = 30.788, *p* = 0.001, χ^2^/*df* = 2.798, *CFI* = 0.968, *SRMR* = 0.029, *RMSEA* = 0.060) (see West et al., 2012). Thus, the relationships between the latent variables are partially confirmed (see also del Arco et al., 2021).

### 4.1. Relationships between challenge and threat appraisals and achievement emotions

Only significant paths are reported. Appraisal of challenge contributed positively to pleasant emotions (γ = 0.383), whereas appraisal of threat contributed positively to unpleasant emotions (γ = 0.360), i.e., higher appraisals of threat implied a higher frequency of unpleasant emotions. Challenge appraisal and threat appraisal were negatively related to each other (γ = −0.316). For the pleasant achievement emotions, the overall mean in the sample was slightly above the scale mean with *M* = 2.84 (*SD* = 0.71) indicating a medium level of pleasant achievement emotions. For appraisal of challenge, the mean in the sample was *M* = 3.48 (*SD* = 0.81), which is above the scale mean of 2.5 (scale ranging from 1 = low to 5 = high). With a mean of *M* = 1.98 (*SD* = 0.87) for appraisal of threat, it was below the scale mean of 2.5 (see Table 1). With *R*^2^ = 0.235 for unpleasant emotions and *R*^2^ = 0.521 for pleasant emotions, a medium respectively larger part of the variance could be explained by appraisals and antecedents of learning.

### 4.2. Relationships between achievement emotions and academic performance

Unpleasant emotions were directly and negatively related to academic performance (γ = −0.200), i.e., higher appraisals implied worse performance. For the unpleasant achievement emotions, the mean in the sample was *M* = 2.16 (*SD* = 0.79), which is slightly below the scale mean of 2.5 (scale ranging from 1 = low to 5 = high). No significant relationship between pleasant emotions and academic performance was found. With *R*^2^ = 0.044 only a small part of the variance of academic performance could be explained by the variables in the SEM.

### 4.3. Personal characteristics, gender, and learning resources

Gender was directly related to proneness to anxiety (γ = −0.225), to unpleasant emotions (γ = −0.072), as well as to academic performance (γ = −0.116), i.e., females showing higher proneness to anxiety and higher levels of unpleasant emotions. The direct relationship between gender and academic performance (γ = −0.116) would indicate better academic performance of women but this relationship is outweighed by the path from gender to unpleasant emotions and worse academic performance in case of more unpleasant emotions plus by the path from gender to proneness to anxiety, threat appraisals, unpleasant emotions, and performance. Additionally, a *t*-test was carried out to investigate differences between female and male students’ achievements. No differences between men and women were found (*T*(312) = 1.289, *p* = 0.198; *M* (Men) = −0.05; *M* (Women) = 0.11).

Proneness to anxiety was negatively related to academic self-concept (γ = −0.355), learning resources (γ = −0.202), and challenge appraisals (γ = −0.105); it was positively related to unpleasant emotions (γ = 0.349) and threat (γ = 0.441). Surprisingly, a positive relationship was found between proneness to anxiety and pleasant emotions, suggesting that higher levels of anxiety are associated with increased experience of positive emotions (γ = 0.150). Academic self-concept was positively related to learning resources (γ = 0.198), to challenge (γ = 0.119), positive emotions (γ = 0.154) and negatively to threat (γ = −0.106) and unpleasant emotions (γ = −0.122). Learning resources were additionally positively related to challenge (γ = 0.261) and pleasant emotions (γ = 0.148) and negatively to threat (γ = −0.164) and unpleasant emotions (γ = −0.097).

For proneness to anxiety, the mean in the sample was *M* = 2.47 (*SD* = 0.97). With a mean of *M* = 4.85 (*SD* = 0.97) for academic self-concept, it was above the scale mean of 3.5 (scale ranging from 1 = low to 7 = high). For the learning resources, the overall mean was *M* = 3.89 (*SD* = 0.60), which was above the scale mean of 2.5 (scale ranging from 1 = low to 5 = high).

## 5. Discussion

### 5.1. Role of challenge and threat appraisals for learning and performance

The results of the structural equation model emphasize the merit of challenge and threat appraisals for students’ emotional experiences in learning and exam preparation.

Threat appraisals, e.g., assessments concerned with doubts about one’s chances to overcome hurdles, were the strongest predictor for unpleasant emotional experiences during learning. These results are in accordance with the biopsychosocial model of challenge and threat (Blascovich and Tomaka, 1996; Seery, 2011, 2013; Wormwood et al., 2019) as well as with previous research (Diotaiuti et al., 2023) suggesting that threat appraisals are linked to distinct patterns of unpleasant emotions. The SEM revealed a pathway connecting the antecedent variables for learning (proneness to anxiety, self-concept, resources) to threat appraisals, then further to unpleasant emotions and ultimately to performance.

Challenge appraisals were the strongest predictor for pleasant emotional experiences during learning and exam preparation. Positive assessments and beliefs of control contributed to positive emotional learning experiences, as it would have been also predicted by the biopsychosocial model of challenge and threat (Blascovich and Tomaka, 1996; Seery, 2011, 2013; Wormwood et al., 2019) and by control-value theory (Pekrun, 2006). Antecedents of learning were related to challenge appraisals: a positive self-concept and access to learning resources increased assessments of control over the situation. In contrast, higher levels of proneness to anxiety reduced such assessments. Contrary to theoretical assumptions, challenge appraisals did not contribute to academic performance. They are only indirectly related to performance via correlation with threat appraisals.

Challenge and threat appraisals exhibited a moderate correlation. However, challenge appraisals displayed no association with unpleasant emotional experiences, while threat appraisals also showed no connection with pleasant emotional experiences.

### 5.2. Role of personal characteristics and learning resources in the learning process

Based on former research, academic self-concept, proneness to anxiety, and access to material and temporal learning resources were recorded as antecedents of learning. Furthermore, gender was recorded.

Proneness to anxiety can be described as a tendency to generally perceive stressful situations as dangerous or threatening (Endler and Kocovski, 2001). Given the difficult circumstances posed by the COVID-19 situation, with its severe restrictions and obstacles for learning, recording proneness to anxiety as a learning-related variable was more or less evident. Within the structural equation model (SEM), this variable demonstrates a dual linkage with unpleasant emotions, by not only amplifying threat appraisals but also establishing a strong direct association with unpleasant emotional experiences. Similar to previous research, students prone to anxiety experienced more unpleasant emotions while preparing for exam (Macher et al., 2012; Alemany-Arrebola et al., 2020; Paechter et al., 2022). According to the cognitive-interference approach highly anxious individuals divide their attention between task-irrelevant thoughts such as self-evaluation and worry, and task-relevant thoughts such as problem-solving. Therefore, anxiety can consume a portion of the processing capacity that would be needed for task performance, in a learning situation as well as in an exam (Eysenck et al., 2007; Macher et al., 2013). As expected, proneness to anxiety also had a small contribution to challenge appraisals, decreasing the perception of controllability of the learning situation.

Rather unexpectedly, proneness to anxiety exhibited a positive correlation with a higher frequency of positive emotions. This finding can be explained by the dual impact anxiety may have on learning. In certain instructional contexts, students who fail to invest sufficient effort and time in their learning endeavors face severe consequences, such as exam failure. Proneness to anxiety may accentuate the severity and unpleasantness of these consequences while also strengthening positive extrinsic motivation for achievement, driven by the intention to avoid failure (Macher et al., 2015). Consequently, this may contribute to learning effort and positive emotions during the learning phase, such as feelings of relief or pride in one’s learning efforts. Likewise, Pekrun et al. (2002) suggest that test-related anxiety may have a positive association with a specific positive emotion known as test relief. In a similar vein, students who actively engaged with the learning situation and successfully overcame internal obstacles, such as anxiety, might have experienced a sense of relief. This actually desired effect of anxiety on pleasant achievement emotions warrants further investigation and should receive greater attention in future research.

Academic self-concept also holds significant importance in learning processes. It took the function of an antagonist to anxiety, as there was a negative correlation between these variables. Academic self-concept acts as a resource that is associated with a reduction in the intensity of threat appraisals. While academic self-concept also has the potential to alleviate unpleasant achievement emotions, it is important to note that anxiety susceptibility remains a more influential factor contributing to such negative emotions.

Learning resources were also positively related to academic self-concept. This is consistent with previous studies which see learning resources as important precursors for academic self-concept (Thomas and Gadbois, 2007; Macher et al., 2012, 2013; Zhou et al., 2015; Bringula et al., 2021). Furthermore, students who perceived their learning resources as adequate experienced more pleasant emotions while preparing for and appraised the learning situation during the COVID-19 pandemic as challenging, rather than threatening.

The structural equation model shows that gender was directly related to proneness to anxiety, unpleasant emotions as well as to academic performance. Comparing to men, women were more prone to anxiety (similar to Macher et al., 2012; Núñez-Peña et al., 2016; Alemany-Arrebola et al., 2020; Bravo et al., 2022; Paechter et al., 2022), they experienced more unpleasant emotions [similar to Pekrun et al. (2017), Fierro-Suero et al. (2022)] while showing better academic performance then men [similar to Zile et al. (2021)]. As it will be described in the next section, gender and academic performance are intertwined in a two-fold way.

### 5.3. Direct contributors to performance, the role of unpleasant emotions and the role of gender

Only two variables contributed directly to academic achievement, experiences of unpleasant achievement emotions and gender.

Unpleasant achievement emotions (like anxiety, anger) are related to dysfunctional cognitions like rumination or worry in learning or surface learning strategies (Macher et al., 2012, 2013). Often, they are accompanied by the learners’ impressions of a lack of control over their learning processes (Pekrun et al., 2007; Macher et al., 2013). According to control-value theory such effects are even stronger if the outcomes are of high value and importance for a learner (Pekrun et al., 2007). As a consequence, one may assume that students with unpleasant emotional experiences in exam preparation were less able to build up an adequate knowledge base and were less prepared for the exam.

For gender, a two-fold effect was found in which positive and negative relations to academic performance outweighed each other. The relation to academic performance indicates an advantage of female over male students. However, men and women did not differ in their academic performance. Women were more prone to anxiety and unpleasant emotions, two variables that were indirectly and directly related with worse performance. Similar effects of gender were found in research on domain-specific anxiety in the subject statistics (Macher et al., 2012, 2013) in which women were impaired by anxiety but exhibited better time-management strategies and less procrastination. According to Macher et al.’s (2012; 2013) studies, the results were constrained within the domain of statistics. However, when combined with the findings of our current study, these results suggest a more extensive and all-encompassing influence.

## 6. Future directions and implications for instruction

The COVID-19 pandemic can be considered as a difficult life circumstance which had a negative psychological impact on university students by threatening their well-being, life satisfaction as well as learning and achievement in academic context. Our study emphasizes the importance of a holistic approach when facing such difficult life circumstances. Universities should take in account an individual learner’s antecedents for learning, appraisals of the specific difficult life circumstance as well as experiences of learning emotions when creating programs to support learning and achievement at universities or when counseling university students.

The structural equation modeling (SEM) analysis suggests that unpleasant achievement emotions serve as a critical variable and significant obstacle to academic performance. Given their connection to personal factors and appraisals, the results indicate various recommendations to mitigate these negative emotions in the learning process. Nevertheless, it is of equal importance that the recommendations encompass fostering positive emotional experiences throughout the learning process. A key is to enhance controllability over learning outcomes (Paechter et al., 2022). For instance, the following strategies can be employed to enhance controllability and reduce anxiety:

*Support for self-concept and providing resources.* Providing for sufficient and adequate resources for students is an important foundation for successful learning (Luttenberger et al., 2018a; Aldhahi et al., 2022; Ramos-Pla et al., 2023). Resources cover material as well as temporal resources. Connected with temporal resources and positive academic performance is also how students manage their resources, e.g., efficient use of temporal resources and avoidance of procrastination (Macher et al., 2013; Aldhahi et al., 2022). Likewise, the academic self-concept can be considered a valuable resource that goes beyond mere knowledge and also includes motivational components. Therefore, educators are advised to promote the development of a positive (yet realistic) academic self-concept (Wang and Yu, 2023). In the context of adverse learning situations, careful selection of the suitable learning and evaluation strategies by educators as well as investing time in communication with students are recommended (Ramos-Pla et al., 2022).

*Support in a positive, yet realistic appraisal of a learning situation.* It is important for learners to realistically assess a learning situation and recognize both obstacles and opportunities for learning. Negative attitudes and characteristics such as anxiety can result in an overly pessimistic perception of the learning environment, which does not align with reality. Here, trainings for learners could be useful. For instance, Perchtold et al. (2018) conducted research on training students to develop realistic evaluations of the learning situation and promote a positive attitude toward learning through re-appraisal techniques. And of course, instructors obtain an important task. In both online and face-to-face settings, they can provide support by offering personalized feedback that links learning achievements to students’ own efforts and performance (Schweizer et al., 2001; Paechter et al., 2020).

*Tailored support for different groups of learners.* The SEM results point to a two-fold relationship between gender and performance. On the one hand, the SEM pointed at deficits of women in learning as well as at advantages. In studies by Macher et al. (2012, 2013) such a two-fold effect in the domain statistics was caused by women’s better time-management while at the same time exhibiting more anxiety in the subject. Taken together, the findings of these studies recommend further investigation into this dual impact, with a specific focus on identifying the behaviors that facilitate learning among women.

While not exhaustive, this list of strategies aims to highlight crucial factors in counteracting unpleasant emotions and promoting pleasant emotions in the learning process. These factors include reducing anxiety, cultivating internal resources, providing external resources, and enhancing controllability.

## 7. Limitations

The present study contributes to research on learning and performance in academic context during the adverse life events. Nevertheless, there are some limitations that need to be addressed.

First, the survey part of the study was cross-sectional. Students’ attitudes, dispositions, appraisals, and achievement emotions were recorded at one point in time, shortly before the exam. In the circumstances of the current study, it was not possible to measure these variables at several points of time. In order to achieve greater insight into causal relations, future studies should investigate these variables in a longitudinal design. Next, our sample were students from predominantly female field of study. Some studies show that dominance of one gender in a specific study field has been related to the gender differences in academic performance (for systematic review see Wang and Yu, 2023). Furthermore, it should be noted that the generalizability to a wider population may be limited due to the specific sample. The findings presented in this study are most directly applicable to students in Psychology and Education. Future research should examine if the relationships between current variables would remain the same for other fields of study as well as for predominantly male and for gender-neutral fields of study.

## Data availability statement

The raw data supporting the conclusions of this article will be made available by the authors, without undue reservation.

## Ethics statement

The study was performed in accordance with the 1964 Declaration of Helsinki and the American Psychological Association’s Ethics Code. The Ethics Committee of the University Graz had approved the study (GZ. 39/78/63 ex 2016/17). All participants gave written consent to participate in the study and to confirm that their data were used in an empirical study.

## Author contributions

SM: Conceptualization, Formal analysis, Investigation, Methodology, Writing – original draft, Writing – review and editing. DM: Formal analysis, Methodology, Writing – review and editing. SH: Conceptualization, Investigation, Writing – review and editing. MP: Conceptualization, Investigation, Supervision, Writing – original draft, Writing – review and editing.
